# Impact of metabolically healthy obesity on the risk of incident gastric cancer: a population-based cohort study

**DOI:** 10.1186/s12902-019-0472-2

**Published:** 2020-01-20

**Authors:** Yoshitaka Hashimoto, Masahide Hamaguchi, Akihiro Obora, Takao Kojima, Michiaki Fukui

**Affiliations:** 1 Department of Endocrinology and Metabolism, Kyoto Prefectural University of Medicine, Graduate School of Medical Science, 465 Kajii-cho, Kawaramachi-Hirokoji, Kamigyo-ku, Kyoto, 602-8566 Japan; 2 Department of Gastroenterology, Asahi University Hospital, Gifu, Japan

**Keywords:** Obesity, Metabolically healthy obesity, Metabolic syndrome, Cancer, Gastric cancer

## Abstract

**Background:**

The risk of colon or breast cancer in metabolically healthy obese (MHO) were lower than that in metabolically abnormal obese (MAO). We hypothesized that the risk of incident gastric cancer in MHO is lower than that in MAO.

**Methods:**

This historical cohort study included 19,685 Japanese individuals who received health-checkup programs from 2003 to 2016. Each subject was classified as metabolically healthy (MH) (no metabolic abnormalities) or metabolically abnormal (MA) (one or more metabolic abnormalities), according to four metabolic factors (hypertension, impaired fasting glucose, hypertriglyceridemia and low HDL-cholesterol). Obese (O) or non-obese (NO) was classified by a BMI cutoff of 25.0 kg/m^2^. Hazard ratios of metabolic phenotypes for incident gastric cancer were calculated by the Cox proportional hazard model with adjustments for age, sex, alcohol consumption, smoking and exercise.

**Results:**

Over the median follow-up period of 5.5 (2.9–9.4) years, incident rate of gastric cancer was 0.65 per 1000 persons-years. Incident rate of MHNO, MHO, MANO and MAO were 0.33, 0.25, 0.80 and 1.21 per 1000 persons-years, respectively. Compared with MHNO, the adjusted hazard ratios for development of gastric cancer were 0.69 (95% CI 0.04–3.39, *p* = 0.723) in MHO, 1.16 (95% CI 0.63–2.12, *p* = 0.636) in MANO and 2.09 (95% CI 1.10–3.97, *p* = 0.024) in MAO.

**Conclusions:**

This study shows that individuals with MAO, but not those with MHO, had an elevated risk for incident gastric cancer. Thus, we should focus more on the presence of metabolic abnormalities rather than obesity itself for incident gastric cancer.

## Background

Gastric cancer is a major global health concern and was the third leading cause of cancer death worldwide in 2012 [1] and gastric cancer is the third leading cause of cancer death in 2016 in Japan [2]. Previous meta-analyses showed that obesity was a risk factor for incident gastric cancer, especially gastric cardia cancer [3], although an umbrella review revealed the effect of obesity on gastric cancer was smaller than that on other obesity-related cancers, such as colon and breast cancers [4].

On the other hand, obesity is also known as a risk factor for type 2 diabetes mellitus (T2DM) [5], chronic kidney disease (CKD) [6] and cardiovascular disease (CVD) [7]. The subgroup of individuals with metabolically healthy obesity (MHO)—i.e., obesity without metabolic abnormalities—are knowns as lower risk of T2DM, CKD and CVD than individuals with metabolic abnormalities obese [8–11]. However, these studies also revealed that individuals with the MHO phenotype were at higher risk of T2DM, CKD and CVD than individuals with metabolically healthy non-obese [8, 10, 11]. In addition, there is accumulating evidence that metabolically abnormal obesity (MAO), but not MHO, confers an elevated risk of incident colon cancer [12] and breast cancer [13]. The association between gastric cancer and obesity among Japanese population is controversial [14, 15]. These studies did not consider the presence of metabolic abnormalities. In contrast, there is an association between metabolic syndrome and incidence of gastric cancer [16–19]. Thus, we thought that not obesity itself, but the presence of metabolic abnormalities, which often accompany with obesity, have an important meaning for gastric cancer.

To our knowledge, however, no previous studies have clarified the relation between MHO and incident gastric cancer. Thus, the aim of this study was to elucidate the impact of MHO on incident gastric cancer.

## Methods

### Study population

This was an historical cohort study of participants who received a medical health-checkup at Asahi University Hospital (the NAGALA (NAfld in Gifu Area, Longitudinal Analysis) study, Gifu, Japan) [20]. The purpose of medical health-checkup was to promote public health by early detection of chronic diseases and their risk factors and about 60–70% examiners received the examinations, repeatedly; thus, the participants represent apparently healthy individuals. Most of the participants of this medical health-checkup were employees of various companies and local governmental organizations in Gifu, Japan, and their consorts. The medical data of all individuals who agreed to participate in the study were stored in a database after removing all personally identifiable information. For the current study, we used the results of individuals who participated in the health-checkup program for at least one year between 2003 and 2016. The exclusion criteria of this study were as follows: the presence of gastric cancer at baseline examination, missing covariate data (body weight, high-density lipoprotein (HDL) cholesterol, and lifestyle factors) and no follow-up health-checkup programs. Informed consent was obtained from each participant. The study was approved by the ethics committee of Murakami Memorial Hospital and was conducted in accordance with the Declaration of Helsinki.

### Data collection

A self-administered questionnaire was used for gathering the medical history and lifestyle factors of participants [20]. In regard to alcohol consumption, participants were asked the type and amounts of alcoholic beverages consumed per week over the past month, and then the mean ethanol intake per week was estimated [21]. For smoking status, the participants were categorized into three groups: never-, ex- and current smokers. In addition, smoking burden was evaluated by pack-years which were calculated by multiplying the number of cigarette packs smoked per day by the number of years of smoking [22]. For exercise, participants were asked to describe the type, duration and frequency of sports or recreational activities [23]. Based on the results, we defined regular exercisers as the participants who performed any kind of sports activity at least once a week on a regular basis [21]. Body mass index (BMI) (kg/m^2^) was calculated as body weight (kg) divided by height (m) squared. Waist circumference was measured as the abdominal circumference around the navel. Fasting plasma glucose, triglycerides, or HDL cholesterol was measured using the venous blood after an overnight fast. The methods for detecting and diagnosing gastrointestinal cancers were described previously [24]. Because the first standardized questionnaires for gastrointestinal cancers were sent on Jan 1st 2003, we set the study period as Jan 1st 2003 to Dec 31st 2016. The primary endpoint of this study was hazard risk (HR) of MHO for gastric cancer.

### Definitions of metabolic phenotypes

We used body mass index > 25.0 kg/m^2^ to identify the individual with obesity. This value has been proposed as a cutoff for the diagnosis of individual with obesity in Asian people [25] and has often been used in Japan [26, 27]. Four metabolic factors (fasting plasma glucose, triglycerides, HDL cholesterol and blood pressure) were used to divide participants into metabolically healthy or metabolically abnormal subgroups [9]. Impaired fasting plasma glucose and/or diabetes was defined as fasting plasma glucose > 5.6 mmol/L and/or current medical treatment. Hypertension was defined as systolic blood pressure > 130 mmHg and/or diastolic blood pressure > 85 mmHg or current medical treatment. Elevated triglycerides were defined as triglycerides > 1.7 mmol/L or treatment for hyperlipidemia. Low HDL-cholesterol was defined as < 1.0 mmol/L in men and < 1.3 mmol/L in women. When none of these four metabolic factors were present, we defined the participants as metabolically healthy (MH) and when one or more of these four metabolic factors were present, we defined the participants as metabolically abnormal (MA) [28]. Then, participants were categorized at the baseline examination into 4 phenotypes: metabolically healthy non-obesity (MHNO), metabolically healthy obesity (MHO); metabolically abnormal non-obesity (MANO), and metabolically abnormal obesity (MAO).

### Statistical analysis

The study participants were divided into four groups based on metabolic phenotypes. Continuous variables were expressed as the means ± standard deviation or median (interquartile range) and categorical variables were expressed as numbers. The clinical characteristics at baseline examination of the four groups were compared; continuous variables of groups were evaluated by one-way ANOVA and Tukey’s Honestly Significant Difference Test or Kruskal-Wallis Test and Steel-Dwass Test, and categorical variables of groups were evaluated by Pearson’s Chi-Squared Test. Because of the censored cases and inconsistent follow-up duration, we used the Cox Proportional Hazards Model to calculate the HR of the four groups. We considered five potential confounders as covariates: age, sex, alcohol consumption [29], pack-years [30], and exercise [31]. Because alcohol consumption and pack-years were skewed variables, logarithmic transformation was carried out before performing the Cox Proportional Hazard Model analysis.

Furthermore, we used the Cox Proportional Hazards Model to calculate the HR of each metabolic abnormality (hypertension, impaired fasting glucose, hypertriglyceridemia and low HDL-cholesterol).

The statistical analyses were performed using JMP version 13.2 software (SAS Institute Inc., Cary, NC). A *p* value < 0.05 was considered statistically significant.

## Results

**Fig. 1:**
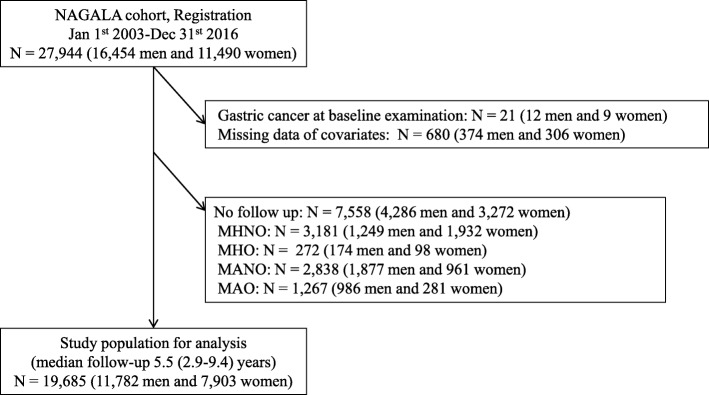
Inclusion and exclusion flow chart. NAGALA, NAfld in Gifu Area, Longitudinal Analysis; MHNO, Metabolically healthy non-obesity; MHO, Metabolically healthy obesity; MANO, Metabolically abnormal non-obesity; MAO, Metabolically abnormal obesity

We included 27,944 participants from the NAGALA database (Fig. 1). Among them, 8259 participants were excluded. Thus, 19,685 participants were eligible for this cohort study. The baseline characteristics of the participants are shown in Table 1. Average age and BMI of this study participants were 45.5 ± 9.5 years old and 22.6 ± 3.3 kg/m^2^ and 59.9% (11,782) were men. In addition, both BMI and metabolic parameters, including blood pressure, fasting plasma glucose, triglycerides and HDL cholesterol, were different among the four metabolic phenotype groups.

**Table 1:** Characteristics of study participants at the baseline examination

	All	MHNO	MHO	MANO	MAO	*p*
N	19,685	8331	653	7276	3425	―
Age, years (mean ± SD)	45.5 ± 9.5	42.6 ± 8.7	43.8 ± 8.3 *	48.3 ± 9.7 *†	47.0 ± 9.0 *†‡	< 0.001
Men, % (n)	59.9% (11,782)	42.0% (3496)	424/229	5088/2188	2774/651	< 0.001
BMI, kg/m^2^ (mean ± SD)	22.6 ± 3.3	20.7 ± 2.1	26.7 ± 1.7 *	22.1 ± 1.9 *†	27.6 ± 2.5 *†‡	< 0.001
Waist circumference, cm (mean ± SD)	78.0 ± 9.6	72.3 ± 7.0	86.9 ± 6.0 *	77.9 ± 6.8 *†	90.6 ± 7.2 *†‡	< 0.001
SBP, mmHg (mean ± SD)	117.5 ± 16.3	108.0 ± 10.7	116.1 ± 8.9 *	122.2 ± 16.2 *†	130.8 ± 15.2 *†‡	< 0.001
DBP, mmHg (mean ± SD)	73.7 ± 11.2	67.2 ± 7.8	72.6 ± 6.7 *	76.9 ± 11.0 *†	82.6 ± 10.1 *†‡	< 0.001
FPG, mmol/L (mean ± SD)	5.4 ± 0.9	5.0 ± 0.3	5.1 ± 0.3 *	5.6 ± 1.0 *†	6.0 ± 1.3 *†‡	< 0.001
Triglycerides, mmol/L (median (interquartile range))	0.8 (0.5–1.2)	0.6 (0.4–0.8)	0.8 (0.6–1.2) *	1.0 (0.6–1.5) *†	1.3 (0.9–1.9) *†‡	< 0.001
HDL cholesterol, mmol/L (mean ± SD)	1.4 ± 0.4	1.6 ± 0.4	1.4 ± 0.3 *	1.3 ± 0.4 *†	1.2 ± 0.3 *†‡	< 0.001
Exercise	16,111/3574	81.4% (6781)	540/113	5911/1365	2879/546	0.002
Smoking status						< 0.001
Never- smoker, % (n)	53.2% (10,480)	65.0% (5414)	53.0% (346)	45.9% (3342)	40.2% (1378)	
Ex-smoker, % (n)	22.4% (4405)	16.0% (1331)	23.4% (153)	26.1% (1897)	29.9% (1024)	
Current smoker, % (n)	24.3% (4776)	16.5% (1375)	23.6% (154)	27.9% (2029)	29.7% (1018)	
Smoking burden, pack-years (median (interquartile range))	0 (0–305)	0 (0–120)	0 (0–300) *	50 (0–420) *†	150 (0–460) *†‡	< 0.001
Alcohol consumption, g/wk. (median (interquartile range))	4.2 (0–90)	1 (0–54)	1 (0–66) *	12 (0–126) *†	12 (1–126) *†	0.070

*MHNO* Metabolically healthy non-obesity; *MHO* Metabolically healthy obesity; *MANO* Metabolically abnormal non-obesity; *MAO* Metabolically abnormal obesity; *BMI* Body mass index; *SBP* Systolic blood pressure; *DBP* Diastolic blood pressure; *FPG* Fasting plasma glucose; *HDL* High-density lipoprotein. Data are the % (number), mean ± standard deviation, or median (interquartile range). The analyses of continuous variables to assess differences among the four groups were performed using one-way ANOVA or Kruskal-Wallis Test, followed by Tukey’s Honestly Significant Difference Test or Steel-Dwass Test. The analyses of categorical variables among the four groups were determined by Pearson’s Chi-Squared Test. *, *p* < 0.05 vs. MHNO; †, *p* < 0.05 vs. MHO; and ‡, *p* < 0.05 vs. MANO

Over the median follow-up period of 5.5 (2.9–9.4) years, incident rate of gastric cancer was 0.65 per 1000 persons-years. Incident rate of MHNO, MHO, MANO and MAO were 0.33, 0.25, 0.80 and 1.21 per 1000 persons-years, respectively.

The results of the Cox proportional hazard model are shown in Table 2 and Additional file 1: Table S1. Compared with the MHNO phenotype, the MAO phenotype (adjusted HR 2.09, 95%CI 1.10–3.97, *p* = 0.024) was associated with a higher risk for development of gastric cancer after adjusting for covariates, whereas the MHO phenotype (adjusted HR 0.69, 95%CI 0.04–3.39, *p* = 0.723) was not.

**Table 2:** Hazard ratio of metabolic phenotype for incident gastric cancer

	MHNO	MHO	MANO	MAO
Person-year, n	51,846.1	3949.65	44,011.9	20,583.1
Incident cases, n	17	1	35	25
Incidence rate per 1000 person-years	0.33	0.25	0.80	1.21
Crude model	Ref	0.77 (0.04–3.77), *p* = 0.797	2.43 (1.38–4.45), *p* = 0.002	3.72 (2.02–7.00), *p* < 0.001
Model 1	Ref	0.68 (0.04–3.32), *p* = 0.691	1.19 (0.66–2.23), *p* = 0.567	2.16 (1.14–4.09), *p* = 0.018
Model 2	Ref	0.69 (0.04–3.39), *p* = 0.723	1.16 (0.63–2.12), *p* = 0.636	2.09 (1.10–3.97), *p* = 0.024

*MHNO* Metabolically healthy non-obesity; *MHO* Metabolically healthy obesity; *MANO* Metabolically abnormal non-obesity; *MAO* Metabolically abnormal obesity; *CI* Confidence interval; *Log* logarithmic. Model 1 was adjusted for age and sex. Model 2 was adjusted for age, sex, exercise habit, log (alcohol consumption + 1) and log (pack-year + 1)

Furthermore, presence of impaired fasting plasma glucose and/or diabetes, hypertension and elevated triglycerides were associated with incident gastric cancer (Table 3).

**Table 3:** Hazard ratio of each metabolic phenotype for incident gastric cancer

	Impaired fasting plasma glucose and/or diabetes (−)	Impaired fasting plasma glucose and/or diabetes (+)
Person-year, n	85,934.94	34,455.79
Incident cases, n	38	40
Incidence rate per 1000 person-years	0.44	1.16
Crude model	Ref	2.67 (1.71–4.17), *p* < 0.001
	Hypertension (−)	Hypertension (+)
Person-year, n	91,735.7	28,655
Incident cases, n	46	32
Incidence rate per 1000 person-years	0.50	1.12
Crude model	Ref	2.28 (1.44–3.56), *p* < 0.001
	Elevated triglycerides (−)	Elevated triglycerides (+)
Person-year, n	104,572	15,818.4
Incident cases, n	62	16
Incidence rate per 1000 person-years	0.59	1.01
Crude model	Ref	1.69 (0.94–2.85), *p* = 0.077
	Low HDL-cholesterol (−)	Low HDL-cholesterol (+)
Person-year, n	93,354.8	27,036
Incident cases, n	55	23
Incidence rate per 1000 person-years	0.59	0.86
Crude model	Ref	1.41 (0.85–2.26), *p* = 0.180

*HDL* High-density lipoprotein

## Discussion

This cohort study of apparently healthy Japanese people is the first to reveal an association between MHO and incident gastric cancer. This study shows that individuals with MAO, but not those with MHO, had an elevated risk for incident gastric cancer. In addition, the presence of impaired fasting plasma glucose and/or diabetes, and hypertension were associated with elevated risk incident gastric cancer.

Obesity was a risk factor for incident gastric cancer [3], although the effect of obesity on gastric cancer was smaller than that on other obesity-related cancers. Previous studies revealed that the risk of incident colorectal cancer [12] and incident breast cancer [13], both of which have been shown to be related to obesity [4], was not high in subjects with MHO. In addition, another study revealed that the risk of obesity-related cancer in MHO was lower than that in MAO [32]. In fact, previous studies revealed the association between metabolic syndrome and incidence of gastric cancer [16–19].

As to why MAO, but not MHO, was associated with a higher risk of incident gastric cancer, there were several possible explanations. It has been reported that metabolic syndrome is associated with gastric cancer [16–19]. In this study, we showed that the presence of metabolic abnormalities, especially impaired fasting plasma glucose and/or diabetes and hypertension, were associated with gastric cancer, which was same as previous studies [33, 34]. Inflammation, as represented by elevation of the pro-inflammatory cytokines tumor necrosis factor-α (TNF-α), interleukin-6 (IL-6), and monocyte chemoattractant protein-1 (MCP-1), is known to be closely associated with not only obesity [35], but also the metabolic abnormalties, including impaired fasting plasma glucose and hypertension [36]. Inflammation leads to the development of gastric cancer by stimulating proliferation and inhibiting apoptosis of human gastric cancer cells [37]. Formation of reactive oxygen species (ROS), by formation of advanced glycation end products [38], leads to DNA damage and development of gastric cancer. In addition, tumor cell progression is stimulated by enhancing the mTOR signaling pathways through an increase in insulin-like growth factor 1 (IGF-1) [39]. On the other hand, it has been reported that the levels of inflammation and IGF-1 in MHO were lower than those in MAO [40, 41]. Collectively, these results could explain why the MAO phenotype, but not the MHO phenotype, was associated with a higher risk of incident gastric cancer.

Some limitations of our study should be noted. First, there was a possibility of selection bias, because we only included the participants who were re-examined in the health-checkup program. There is a possibility that there is a characteristic difference between the participants who were re-examined in the health-checkup program and those who did not. Second, we did not have data on *H. pylori* infection, which is known to pose a risk for gastric cancer [42]. In fact, many Japanese, especially elderly people, are infected with *H. pylori* [43]. Therefore, the results of this study might have been affected by the status of *H. pylori* infection. Third, we did not have detailed data on gastric cancer according to the anatomic location of the lesion, such as gastric non-cardia cancer and gastric cardia cancer. A previous study revealed that gastric cardia cancer showed a greater association with obesity than non-cardia cancer [1]. Lastly, the generalizability of our study to non-Japanese populations is uncertain.

## Conclusion

In conclusion, our study showed that MAO individuals, not but MHO individuals, had a higher risk of incident gastric cancer. Thus, to prevent future gastric cancer, we should focus more on the presence of metabolic abnormalities rather than obesity itself.

## Supplementary information

**Additional file 1: Table S1.** Hazard ratio of potential confounders for incident gastric cancer.

## Data Availability

The datasets generated during and/or analyzed during the current study are available from the corresponding author on reasonable request.

## Abbreviations

BMI: Body mass index
CKD: Chronic kidney disease
CVD: Cardiovascular disease
HDL: High-density lipoprotein
HR: Hazard ratio
IGF-1: Insulin-like growth factor 1
IL-6: Interleukin-6
MANO: Metabolically abnormal non-obesity
MAO: Metabolically abnormal obesity
MCP-1: Monocyte chemoattractant protein-1
MHNO: Metabolically healthy non-obesity
MHO: Metabolically healthy obesity
ROS: Reactive oxygen species
T2DM: Type 2 diabetes mellitus
TNF-α: Tumor necrosis factor-α
