# Supplementary material for: Impact of metabolically healthy obesity on the risk of incident gastric cancer: a population-based cohort study
Source: BMC Endocr Disord. 2020 Jan 20;20:11. doi: 10.1186/s12902-019-0472-2 (PMC6971909; doi:10.1186/s12902-019-0472-2)
Supplement: Supplementary file 1 — Additional file 1: Table S1. Hazard ratio of potential confounders for incident gastric cancer. [file 12902_2019_472_MOESM1_ESM.docx]

**Table S1. Hazard ratio of potential confounders for incident gastric cancer**

|  | **Crude model** | **Adjusted model** |
| --- | --- | --- |
| Age (years) | 1.13 (1.10-1.15), *p* <0.001 | 1.12 (1.09–1.15), *p* <0.001 |
| Men | 2.65 (1.55-4.82), *p* <0.001 | 0.91 (0.43–1.92), *p* = 0.811 |
| Exerciser | 1.30 (0.73-2.17), *p* = 0.343 | 0.91 (0.53–1.58), *p* = 0.745 |
| Log (pack-year + 1) | 1.27 (1.16-1.38), *p* <0.001 | 1.16 (1.04–1.28), *p* = 0.005 |
| Log (alcohol consumption +1) | 1.17 (1.06-1.30), *p* = 0.002 | 1.04 (0.94–1.16), *p* = 0.412 |

Adjusted model was adjusted for the presence of metabolic phenotypes **(**metabolically healthy non-obesity, metabolically healthy obesity, metabolically abnormal non-obesity and metabolically abnormal obesity).
